# Family Relationships as Modifiable Targets for Caregiver Quality of Life in Hospice Care: A Multicenter Study

**DOI:** 10.3390/curroncol33050301

**Published:** 2026-05-21

**Authors:** In Cheol Hwang, Youn Seon Choi, Hong Yup Ahn, So-Jung Park, Yoo Jeong Lee

**Affiliations:** 1 Department of Family Medicine, Gil Medical Center, Gachon University College of Medicine, Incheon 21565, Republic of Korea; ichwang@gilhospital.com; 2 Palliative Care Center, Korea University Guro Hospital, Seoul 08308, Republic of Korea; younseon@korea.ac.kr; 3 Department of Family Medicine, Korea University Guro Hospital, Seoul 08308, Republic of Korea; 4 Department of Statistics, Dongguk University, Seoul 04620, Republic of Korea; ahn@dongguk.edu; 5 Department of Hospice and Palliative Service, National Cancer Center, Goyang 10408, Republic of Korea

**Keywords:** family caregiver, family relationships, hospice care, palliative care, quality of life

## Abstract

Family caregivers play a critical role in supporting patients with advanced cancer, yet their quality of life (QoL) is often adversely affected and remains insufficiently addressed in routine care. Although family relationships have been widely recognized as important in the caregiving context, their specific domains have rarely been examined in relation to caregiver outcomes. This study aimed to examine the associations between distinct domains of family relationships and caregiver QoL. A total of 170 caregivers were recruited from nine hospice units in Korea between September 2021 and March 2024. for this multicenter study. The Family Relationship Assessment Scale (FRAS) and the Korean version of the Caregiver QOL Index-Cancer (CQOLC-K) were used to assess family relationships and caregiver QoL, respectively. Multivariate regression analyses were performed to evaluate the associations between specific domains of family relationships and caregiver QoL. Family relationship domains were differentially associated with caregiver QoL. Overall family relationship scores were positively associated with QoL (β = 0.30, *p* = 0.004), while family conflict showed the strongest negative association (β = −1.03, *p* = 0.001). In contrast, family support was associated with better positive adaptation (β = 0.24, *p* = 0.027). The associations between family relationships and QoL were more pronounced among vulnerable caregivers, including those who were younger, unemployed, had lower social support or resilience, or were dissatisfied with care. Family relationships, particularly conflict and support, are important correlates of caregiver QoL. Incorporating the assessment of family relationship domains helps identify caregivers at increased risk and informs the development of more family-centered supportive approaches in palliative oncology care.

## Simple Summary

Family caregivers play a crucial role in supporting patients with advanced cancer, yet their quality of life is often overlooked in clinical practice. This study examined how different aspects of family relationships—such as support, conflict, and togetherness—affect caregivers’ well-being in hospice care settings. We found that family conflict was strongly associated with poorer quality of life, whereas family support was linked to better coping. These effects were particularly pronounced among caregivers with fewer psychological or social resources. Our findings suggest that assessing family relationships helps clinicians identify caregivers at higher risk and guide more effective, family-centered support strategies in palliative oncology care.

## 1. Introduction

Family caregivers (FCs) play a central role in the care of patients with advanced cancer receiving hospice care, providing substantial physical, emotional, and practical support throughout the disease trajectory [1]. While their contributions are essential to maintaining patient quality of life (QoL), caregiving is frequently associated with significant psychological distress, physical strain, and socioeconomic burden [2]. Importantly, caregiver well-being is not only an outcome in itself but also a determinant of patient outcomes, including symptom control, healthcare utilization, and end-of-life experience [3]. Accordingly, hospice frameworks emphasize that both patients and their families should be considered recipients of care.

However, despite this recognition, systematic assessment and targeted support for FCs remain insufficiently integrated into routine oncology practice [4,5]. In particular, family relationships—an essential component of the caregiving context—are rarely assessed in a structured manner in hospice care settings. Although FCs often experience substantial distress, sometimes exceeding that of patients, they continue to receive comparatively limited attention from healthcare professionals [6,7,8]. Therefore, FCs should be recognized not only as contributors to care but also as recipients requiring comprehensive assessment and support.

Family relationships are known to influence mental health and QoL in cancer care [8]. However, evidence regarding their association with caregiver QoL remains limited and inconsistent [9,10]. Moreover, prior studies have largely treated family relationships as contextual variables rather than as potentially modifiable targets for intervention. From a clinical perspective, identifying such modifiable psychosocial factors is critical for developing structured care strategies. Family relationships are also shaped by cultural context. In East Asian settings, where caregiving is strongly embedded within family systems influenced by Confucian values, both supportive and conflictual dynamics may be amplified, underscoring the need for culturally informed evaluation.

Therefore, this study aimed to examine the associations between family relationship domains and caregiver QoL and to explore how specific components—support, conflict, and togetherness—are related to caregiver outcomes among FCs of patients with advanced cancer receiving hospice care. We hypothesized that family relationship domains would be associated with caregiver QoL among family caregivers of patients with advanced cancer. We further expected that these associations would vary according to caregiver characteristics and caregiving contexts, reflecting the heterogeneity of caregiving experiences. By focusing on distinct domains of family relationships, this study seeks to provide clinically relevant insights that inform more family-centered approaches in palliative oncology care.

## 2. Materials and Methods

### 2.1. Study Design and Participants

This multicenter study was conducted in nine hospice care units in the Republic of Korea from September 2021 to March 2024. This study was designed to identify clinically actionable psychosocial factors that could be integrated into routine hospice care assessment and intervention. The inclusion criteria encompassed primary FCs aged ≥20 years who were identified by the patient or family as the main individual responsible for providing care and who devoted the majority of their time to caregiving duties without formal compensation. Individuals with significant cognitive impairment or communication difficulties that precluded completion of the questionnaire were excluded. Researchers and trained assistants explained the study’s purpose, obtained informed consent, and administered self-reported questionnaires to the participants. The self-administered questionnaire assessed FCs’ actual care burden, social support, quality of care, psychological resilience, emotional distress, QoL, and family relationships. To ensure the completeness of the data, responses were reviewed immediately and missing data were promptly addressed. All participants provided written informed consent, and the study was approved by the institutional review boards of all participating institutions. Given the observational multicenter design of the study, a formal sample size calculation was not performed.

### 2.2. Primary Variables

The QoL of FCs was assessed using the Korean version of the Caregiver QOL index-Cancer (CQOLC-K), a validated and comprehensive tool designed for this purpose [11]. It comprises 35 items that evaluate various dimensions of QoL, including physical, emotional, family, and social functioning. The subscales of the CQOLC-K include burdensomeness, disruptiveness, positive adaptation, and financial concerns. Respondents expressed their responses to each item on a 5-point Likert-type scale ranging from 0 (“not at all”) to 4 (“very much”). Cumulative scores were derived by adding individual item scores, with higher scores indicating enhanced QoL. The instrument has demonstrated good internal consistency (Cronbach’s alpha = 0.90).

Family relationships were evaluated using the Korean Family Relationship Assessment Scale (FRAS), a validated and user-friendly 15-item instrument designed to assess the perceived dynamics of familial relationships as reported by family members [12]. The FRAS demonstrated good internal consistency, with Cronbach’s alpha coefficients of 0.89 for the total scale, 0.87 for family support, and 0.77 for both family conflict and togetherness. It assesses three factors: family support, family conflict, and family togetherness. Each item is scored on a 5-point Likert-type scale ranging from 1 (“strongly disagree”) to 5 (“strongly agree”). Higher total scores on the FRAS indicate more favorable perceptions of family relationships, signifying greater family support and togetherness and lower levels of family conflict.

### 2.3. Covariates

The participants’ clinical information included demographic characteristics (such as age, sex, relationship with the patient, educational level, marital status, current employment status, and religion). Additionally, questionnaires for FCs queried about caregiving environment, psychological resilience, and emotional distress. These variables were included to account for potential confounding factors and to explore differences across caregiver subgroups.

The questions regarding the caregiving environment, included questions on actual care burden (hours spent per day and visiting days per week and month during caregiving), perceived social support, and the quality of care provided. The extent of social support for the FCs was assessed using the Korean version of the Medical Outcome Study Social Support Survey (MOS-SSS), a validated tool comprising 19 items appraised on a 5-point Likert scale. It encompasses four aspects of social support: emotional/informational, tangible, affectionate, and positive social interactions [13]. Higher scores indicate a greater level of perceived social support. Cronbach’s alpha coefficient of this tool was 0.97, demonstrating internal consistency. Quality of care was assessed using the Quality Care Questionnaire-End of Life (QCQ-EOL), a validated tool consisting of 16 items appraised on a 4-point Likert scale [14]. Higher total scores represent a higher perceived quality of care, ranging from 16 to 64. The internal consistency of this instrument was acceptable, with Cronbach’s alpha coefficients ranging from 0.73 to 0.89.

Psychological resilience was assessed using the Korean version of the Connor-Davidson Resilience Scale (CD-RISC), a validated tool designed to measure resilience [15]. The CD-RISC comprises 25 items rated on a 5-point scale ranging from 0 (“not true at all”) to 4 (“true nearly all of the time”) based on the applicability of each item to them over the preceding month. The total score is obtained by summing all the responses, with higher scores indicating greater resilience, and the instrument has demonstrated good internal consistency (Cronbach’s alpha = 0.93).

Emotional distress among FCs was examined using the Korean version of the Hospital Anxiety and Depression Scale (HADS), a validated instrument comprising two subscales that assess anxiety and depression experienced within the preceding week [16]. Each subscale consists of seven items, with ratings ranging from 1 to 3 points per item. Overall HADS scores range from 0 to 21. The instrument has demonstrated good internal consistency, with Cronbach’s alpha coefficients of 0.89 and 0.86 for the anxiety and depression subscales, respectively.

### 2.4. Statistical Analysis

Descriptive statistics for participants’ characteristics are presented as mean ± standard deviation (SD) for continuous variables, and as frequencies and percentages for categorical variables. An independent *t*-test was performed to compare characteristics according to the QoL levels of FCs. Stepwise multivariate regression analysis was used to identify factors associated with the QoL of FCs and multivariate regression analyses were conducted for subgroup analysis and associations between the FRAS subscales and CQOLC. Given the number of potential covariates relative to the sample size, stepwise regression was used as an exploratory approach to identify the most relevant predictors while maintaining model parsimony. The sample size was considered adequate for multivariate regression analyses given the number of predictors included in the model. Statistical significance was set at *p* < 0.05. All the statistical analyses were performed using STATA/MP version 17.0 (Stata Corp., College Station, TX, USA).

## 3. Results

### 3.1. Participant Characteristics and the Distribution of Scores

**Figure 1:**
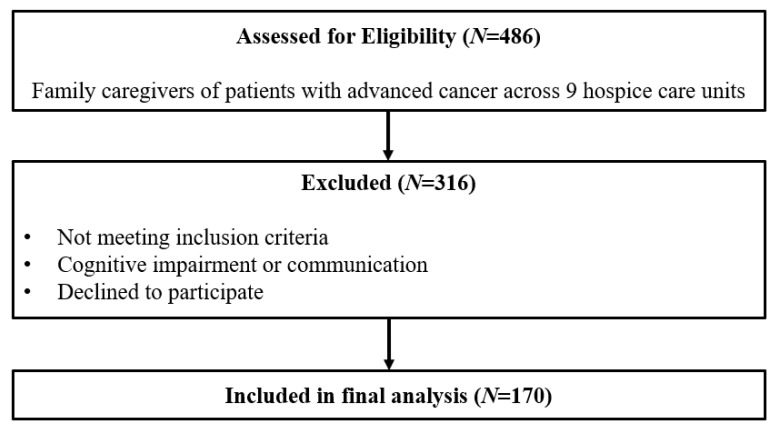
Flow diagram of participant inclusion and analysis.

A total of 486 caregivers were assessed for eligibility, and 170 were included in the final analysis (Figure 1). Table 1 presents the participants’ characteristics and the distribution of scores. FCs in this study had a mean age of 53.7 years and comprised 75.9% females. Approximately 40.6% of the FCs were spouses of patients. On average, the FCs provided intensive care for 18.8 h per day, 5.7 days per week, for a mean duration of 8.2 months in a year.

### 3.2. Factors and Correlations Related to Family Caregivers’ Quality of Life

The factors associated with the QoL of the FCs are listed in Table 2. Higher CQOLC-K scores indicate better quality of life; therefore, negative β coefficients correspond to poorer QoL, whereas positive coefficients indicate better QoL. In a stepwise multivariate regression model, the significant predictors of QoL included satisfactory quality of care (β = 0.31, *p* = 0.019), emotional distress (β = −1.57, *p* < 0.001), and a higher family relationship score (FRAS) (β = 0.30, *p* = 0.004). The final model explained 58.3% of the variance in caregiver quality of life (Adjusted R^2^ = 0.583). Specifically, within family relationships, a high level of family conflict (β = −1.03, *p* = 0.001) was significantly associated with QoL. Family support demonstrated a positive association with positive adaptation (β = 0.24, *p* = 0.027) while family conflict revealed a negative association with disruptiveness (β = −0.35, *p* = 0.013) (Table 3).

### 3.3. Vulnerable Caregiver Subgroups and Family Relationship Dynamics 

Table 4 presents subgroup analysis for the association between familial relationships and QoL, which were performed based on age, employment status, social support, quality of care, and resilience. The cut-off values used in subgroup analyses were based on median values of the study sample. The association remained significant in the subgroups of participants who were younger in age, unemployed, had low social support or resilience, and were dissatisfied with the quality of care; it was not significant in the counterpart groups.

## 4. Discussion

This multicenter study provides evidence that family relational dynamics play a meaningful role in shaping the QoL of FCs of patients with advanced cancer receiving hospice care. Rather than demonstrating a uniform effect of family functioning, our findings indicate that distinct dimensions of family relationships are differentially associated with caregiver outcomes. Specifically, higher levels of family support were associated with better positive adaptation, whereas higher levels of family conflict were associated with greater disruptiveness in caregivers’ daily lives. These results suggest that family relationships do not exert a homogeneous influence on caregiver well-being; instead, different relational domains operate through distinct pathways that can either buffer or exacerbate caregiving burden. Although several sociodemographic and caregiving-related factors were significantly associated with QoL in univariate analyses, they were not retained in the multivariate model, likely reflecting confounding and interrelationships among variables. In particular, the effects of factors such as age and sex may have been attenuated after adjustment for psychological and relational variables, including emotional distress and family relationship domains, which demonstrated stronger associations with caregiver QoL. This domain-specific approach provides a more nuanced understanding of how family dynamics relate to caregiver QoL in hospice care settings and advances current knowledge by highlighting distinct relational mechanisms within palliative oncology care.

Among the family relationship domains examined, family conflict showed the strongest negative association with caregiver QoL. This finding suggests that relational tension within families represents a particularly salient source of stress for FCs in the hospice care context. Family conflict may hinder effective communication, limit the availability of emotional and practical support, and exacerbate caregiving burden, thereby contributing to psychological distress and functional disruption [17,18]. The lack of psychological and practical support—such as emotional and financial assistance—due to family conflict leaves FCs feeling isolated and overburdened, directly impacting their QoL [19]. In contrast, family support appeared to facilitate adaptive coping, potentially by promoting emotional security, shared responsibility, and constructive problem-solving within the caregiving environment. These results indicate that not all aspects of family relationships exert equivalent influence, and that conflict-related dynamics may be especially detrimental to caregiver well-being. Prior studies have shown that caregivers who maintain open communication within families experience lower levels of psychological distress, and that family-centered support strategies help reduce relational strain and improve coping capacity [20,21]. In cultural contexts where familial harmony is emphasized, such as Confucian-influenced societies, these dynamics may be particularly prominent.

Subgroup analyses further revealed that the association between family relationships and caregiver QoL was more pronounced among certain vulnerable caregiver groups. In particular, the influence of family relationships on QoL was stronger among caregivers who were younger, unemployed, reported lower levels of social support or resilience, or were dissatisfied with the quality of care. These factors have been identified as vulnerabilities of FCs in previous studies [22,23,24]. One possible explanation for these findings is that younger caregivers have fewer caregiving experiences or limited external support systems, which could increase their reliance on family relationships as a source of coping [25]. Similarly, unemployed caregivers depend more heavily on family networks in the absence of workplace-based social and practical resources [26]. Lower levels of social support and dissatisfaction with care quality also intensify feelings of isolation and burden, thereby heightening the influence of family dynamics on caregiver well-being. In addition, caregivers with lower resilience have fewer internal coping resources, making them more sensitive to the presence or absence of supportive family relationships [27,28]. These findings suggest that the impact of family relational dynamics is not uniform across all caregivers, but may be amplified in individuals with fewer psychological or social resources. The substantial variability in caregiving duration observed in this study further suggests that the caregiving experience differs across shorter and more prolonged trajectories. In this context, the influence of family conflict on caregiver quality of life varies depending on the duration of caregiving, which was not explicitly examined in the present analysis and warrants further investigation. For these caregivers, family relationships play a critical role in either mitigating or exacerbating caregiving burden. Identifying such high-risk caregiver profiles is therefore be important for prioritizing supportive resources and tailoring family-centered approaches within palliative oncology care.

In this study, the mean total CQOLC score was 70.7, which appears lower than scores reported in Western countries such as Canada and the United States (98.8 and 95.3, respectively), but comparable to findings from other East Asian populations, including Korea and Taiwan (67 and 81.7, respectively) [29,30,31,32]. These differences reflect variations in caregiving expectations and cultural norms across settings. In East Asian contexts influenced by Confucian traditions, caregiving is often regarded as a familial responsibility and moral obligation, which intensifies both the psychological and practical demands placed on caregivers [10,33]. This sociocultural framework is also reflected in the gender distribution of caregivers in the present study, where women comprised the majority of participants. In many Asian societies, caregiving roles are more frequently undertaken by women due to deeply rooted social and familial expectations, which contributes to a disproportionate caregiving burden among female caregivers [34]. Such patterns are associated with greater emotional strain and should be considered when interpreting caregiver experiences and outcome measures. This cultural context amplifies the influence of family relationships on caregiver well-being, particularly when relational conflict is present. Consistent with this, levels of anxiety and depression observed in our study were comparable to those reported in other Eastern populations but higher than those observed in Western studies [35,36]. Together, these findings suggest that cultural context shapes not only caregiving experiences but also the ways in which family relationships influence caregiver outcomes.

Notably, the mean HADS score in this study was 18.9 ± 8.2, indicating a high level of emotional distress among caregivers. This finding suggests that psychological burden is substantial in this population and represents an important underlying factor influencing caregiver QoL [37]. Elevated emotional distress is also likely to interact with family relational dynamics, potentially exacerbating the negative effects of family conflict while diminishing the buffering role of family support [38]. These findings further support the notion that caregiver outcomes are shaped not only by relational contexts but also by underlying psychological vulnerability, highlighting the interconnected nature of emotional distress and family dynamics.

**Figure 2:**
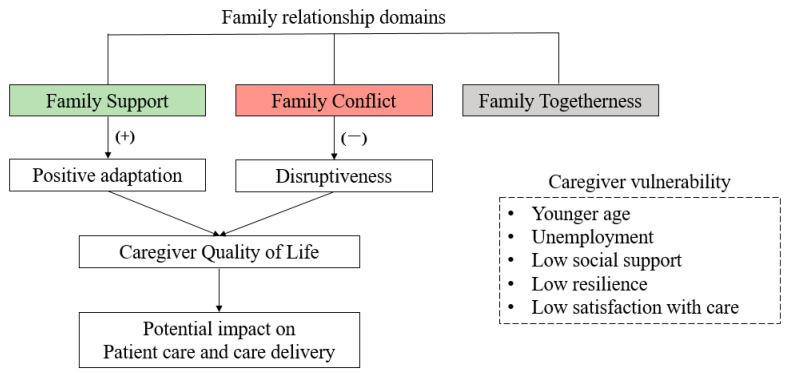
Conceptual framework illustrating the associations between family relationship domains and caregiver quality of life.

Taken together, these findings indicate that family relationships should be considered an important component of caregiver assessment in palliative oncology care. Rather than viewing relational dynamics as background characteristics, systematic evaluation of specific domains—notably family conflict and perceived support—help identify caregivers at increased risk of poor QoL. In clinical practice, incorporating brief assessments of family relationships into routine caregiver evaluations could facilitate earlier recognition of relational strain and guide supportive responses. Such approaches are particularly relevant for caregivers with fewer psychological or social resources, in whom the impact of family dynamics appears to be amplified. Supportive strategies, including facilitated family communication, early psychosocial support, and the involvement of interdisciplinary team members, can address relational challenges and improve caregiver coping. In this context, our findings can be conceptualized as a framework linking family relationship domains, caregiver vulnerability, and caregiver outcomes. which can inform more integrated and family-centered approaches in palliative oncology care (Figure 2). This conceptual illustrates that family relationship domains (support, conflict, and togetherness) are directly associated with caregiver quality of life, while caregiver vulnerability factors modify the strength of these associations. In particular, supportive relationships buffer caregiving burden, whereas conflictual dynamics exacerbate psychological distress and functional disruption. Accordingly, these findings can inform more integrated and family-centered approaches in palliative oncology care.

This study has several limitations that must be considered. First, the inclusion of only Korean FCs restricts the generalizability of the results to other countries. Second, the focus on the FCs of patients with advanced cancer receiving hospice care limits the applicability of the findings to those in-home hospice settings or other care environments. Third, its cross-sectional design permits only the observation of associations, precluding the establishment of causality. Reverse causality cannot be excluded, as lower caregiver quality of life or greater exhaustion contributes to increased family conflict rather than family conflict solely leading to poorer outcomes. The inability to monitor changes in family relationships throughout the illness trajectory also needs to be acknowledged. Additionally, all variables were assessed using self-reported measures, which introduce reporting bias. Although the FRAS was originally validated in Korean college student families, its core domains are relevant to caregiving contexts; however, its use in hospice populations has not been extensively validated and should be interpreted with caution. The cut-off values used in the subgroup analyses were based on the median of the study sample rather than established clinical thresholds, which reduces the generalizability of these findings. The use of stepwise regression can affect the stability of variable selection, and the absence of statistical correction for multiple testing increases the possibility of spurious associations; therefore, these findings should be interpreted with appropriate caution.

Despite these limitations, this study demonstrated the significant influence of family relationships on the QoL of FCs in hospice care settings, along with a subscale analysis of the dimensions of family relationships. This indicates that healthcare professionals need to understand family relationships when delivering hospice care by integrating the dynamics of family relationships into care planning and interventions. By recognizing the challenges that caregivers face, healthcare professionals can provide tailored support systems that address both emotional and practical needs. Longitudinal studies with repeated measurements are essential to confirm the results and investigate how family dynamics change over time. Furthermore, research exploring diverse cultural contexts could yield a more comprehensive understanding of the experiences faced by FCs. Consequently, these insights can lead to improved support for FCs and better outcomes for patients with advanced cancer receiving hospice care.

## 5. Conclusions

This multicenter study highlights that family relationship dynamics are important factors associated with caregiver QoL in patients with advanced cancer receiving hospice care. Among these, family conflict emerged as the most detrimental factor, whereas family support was associated with improved adaptive outcomes. These findings position family relationships as clinically actionable targets rather than passive background factors in palliative oncology care. Routine assessment of family relationship domains facilitates early identification of high-risk caregivers and enables tailored, family-centered interventions, ultimately improving the quality of supportive care for patients and their families in palliative oncology settings.

## Figures and Tables

**Table 1:** Participant characteristics and the distribution of scores (*N* = 170).

		Range	
	Mean ± SD or *N* (%)	Interquartile	Possible
Demographics			
Age (year)	53.7 ± 12.9	44–62	
Female sex	129 (75.9)		
Spouse	68 (40.6)		
College graduation or higher	94 (55.6)		
Married	131 (78.0)		
Currently employed	72 (42.6)		
Professing a religion	92 (54.8)		
Caregiving environment			
Actual care burden			
Hours spent per day in caregiving	18.8 ± 7.6	12–24	1–24
Visiting days per week	5.7 ± 2.0	5–7	1–7
Months in caregiving	8.2 ± 12.2	1–9	
Social support (MOS-SSS total score)	75.4 ± 17.8	61–91	0–100
Quality of care (QCQ-EOL total score)	26.6 ± 8.0	21–32	16–64
Psychological resilience (CD-RISC score)	58.6 ± 17.2	47–69	0–100
Emotional distress (HADS score)	18.9 ± 8.2	12–25	0–42
Family relationship (FRAS score)			
Overall	56.9 ± 10.8	50–65	15–75
Family support	19.2 ± 4.1	16–23	5–25
Family conflict	10.0 ± 3.7	7–12	5–25
Family togetherness	17.8 ± 4.0	15–21	5–25
Quality of life (CQOLC score)			
Total	70.7 ± 18.1	57–83	0–140
Burdensomeness	16.8 ± 8.0	11–23	0–40
Disruptiveness	15.4 ± 5.5	12–19	0–28
Positive adaptation	14.7 ± 4.9	11–18	0–28
Financial concerns	7.7 ± 3.1	6–10	0–12

Data are presented as mean ± SD or *N* (%), unless otherwise specified. Abbreviations: MOS-SSS, Medical Outcome Study Social Support Survey; QCQ-EOL, Quality Care Questionnaire-End of Life; CD-RISC, Connor-Davidson Resilience Scale; HADS, Hospital Anxiety and Depression Scale; FRAS, Family Relationship Assessment Scale; CQOLC, Caregiver Quality of Life Index-Cancer; SD, standard deviation.

**Table 2:** Factors associated with family caregivers’ quality of life.

	Univariate		Stepwise Multivariate	
	β (95% CI)	*p*-Value	β (95% CI)	*p*-Value
Old age (per 1-y increase)	0.26 (0.04–0.47)	0.018		
Female sex	−7.64 (−13.97–−1.32)	0.018		
Frequency of visits (per 1-day increase)	−1.53 (−2.96–−0.10)	0.037		
Greater social support ^a^	0.24 (0.09–0.39)	0.002		
Satisfaction with quality of care ^a^	0.78 (0.43–1.12)	<0.001	0.31 (0.05–0.57)	0.019
High resilience ^a^	0.40 (0.25–0.55)	<0.001		
Emotional distress ^a^	−1.59 (−1.83–−1.36)	<0.001	−1.57 (−1.83–−1.30)	<0.001
Higher family relationship score (FRAS) ^a^	0.63 (0.40–0.87)	<0.001	0.30 (0.09–0.50)	0.004
High family support	1.58 (0.96–2.20)	<0.001		
High family conflict	−1.87 (−2.56–−1.17)	<0.001	−1.03 (−1.61–−0.44)	0.001
High family togetherness	1.30 (0.64–1.96)	<0.001		
			Adjusted R^2^ = 0.583	

β represents regression coefficients for the total quality-of-life score (CQOLC) from the stepwise multivariate linear regression model. Variables listed in Table 1 were considered, and only significant variables were retained in the final model. ^a^ Per 1-point increase in each questionnaire score. Abbreviations: FRAS, Family Relationship Assessment Scale.

**Table 3:** Association between familial relationship domains and quality of life subscales for family caregivers.

	Burdensomeness		Disruptiveness		Positive Adaptation		Financial Concerns	
Per 1-Point Increase	β (95% CI)	*p*-Value	β (95% CI)	*p*-Value	β (95% CI)	*p*-Value	β (95% CI)	*p*-Value
Family support	−0.03 (−0.35–0.30)	0.869	0.17 (−0.09–0.43)	0.198	0.24 (0.03–0.46)	0.027	0.01 (−0.14–0.16)	0.885
Family conflict	−0.13 (−0.49–0.22)	0.463	−0.35 (−0.63–−0.08)	0.013	−0.05 (−0.29–0.19)	0.692	−0.17 (−0.28–0.05)	0.157
Family togetherness	−0.14 (−0.46–0.18)	0.396	0.20 (−0.05–0.46)	0.116	0.17 (−0.05–0.38)	0.134	0.04 (−0.11–0.19)	0.638

β coefficients were derived from multivariate regression models for each quality-of-life subscale, adjusted for 7 covariates, including age, sex, caregiving burden, social support, resilience, emotional distress, and quality of care.

**Table 4:** Subgroup analysis for the association between family relationships and quality of life for family caregivers.

	CQOLC Total Score	
Per 1-Point Increase in Overall FRAS Score	β (95% CI)	*p*-Value
Overall	0.27 (0.01–0.53)	0.046
Young age (<53)	0.48 (0.11–0.84)	0.011
Unemployed	0.48 (0.13–0.82)	0.008
Low social support (MOS-SSS < 75)	0.29 (0.004–0.58)	0.047
Non-satisfied with quality of care (QCQ-EOL < 27)	0.51 (0.04–0.98)	0.035
Low resilience (CD-RISC < 59)	0.46 (0.05–0.87)	0.030

β coefficients were derived from multivariate regression models adjusted for all variables listed in Table 1 within each subgroup. Abbreviations: FRAS, Family Relationship Assessment Scale; CQOLC, Caregiver Quality of Life Index-Cancer; MOS-SSS, Medical Outcome Study Social Support Survey; QCQ-EOL, Quality Care Questionnaire End of Life; CD-RISC, Connor-Davidson Resilience Scale.

## Data Availability

The data presented in this study are not publicly available due to privacy and ethical restrictions but are available from the corresponding author upon reasonable request.
